# Bcl-2 Family of Proteins in the Control of Mitochondrial Calcium Signalling: An Old Chap with New Roles

**DOI:** 10.3390/ijms22073730

**Published:** 2021-04-02

**Authors:** Jordan L. Morris, Germain Gillet, Julien Prudent, Nikolay Popgeorgiev

**Affiliations:** 1 Medical Research Council Mitochondrial Biology Unit, Cambridge Biomedical Campus, University of Cambridge, Cambridge CB2 0XY, UK; jlm96@mrc-mbu.cam.ac.uk; 2 Université de Lyon, Centre de Recherche en Cancérologie de Lyon, U1052 INSERM, UMR CNRS 5286, Université Lyon I, Centre Léon Bérard, 28 rue Laennec, 69008 Lyon, France; germain.gillet@univ-lyon1.fr; 3 Hospices Civils de Lyon, Laboratoire d’Anatomie et Cytologie Pathologiques, Centre Hospitalier Lyon Sud, Chemin du Grand Revoyet, 69495 Pierre Bénite, France

**Keywords:** Bcl-2 proteins, mitochondrial calcium homeostasis, VDAC, IP_3_R, apoptosis, cell migration

## Abstract

Bcl-2 family proteins are considered as one of the major regulators of apoptosis. Indeed, this family is known to control the mitochondrial outer membrane permeabilization (MOMP): a central step in the mitochondrial pathway of apoptosis. However, in recent years Bcl-2 family members began to emerge as a new class of intracellular calcium (Ca^2+^) regulators. At mitochondria-ER contacts (MERCs) these proteins are able to interact with major Ca^2+^ transporters, thus controlling mitochondrial Ca^2+^ homeostasis and downstream Ca^2+^ signalling pathways. Beyond the regulation of cell survival, this Bcl-2-dependent control over the mitochondrial Ca^2+^ dynamics has far-reaching consequences on the physiology of the cell. Here, we review how the Bcl-2 family of proteins mechanistically regulate mitochondrial Ca^2+^ homeostasis and how this regulation orchestrates cell death/survival decisions as well as the non-apoptotic process of cell migration.

## 1. Introduction

Apoptosis is a form of regulated cell death by which complex multicellular organisms orchestrate the regulated removal of unwanted or damaged cells. It is well established that apoptosis plays critical roles in development, tissue homeostasis and the response to cellular stress [1]. Aberrations in the apoptotic program contribute to the aetiology of a broad range of human pathologies including cancer and neurodegenerative diseases [2,3].

Mitochondria play a central role in apoptosis execution. Actually, these genuine intracellular powerhouses contain in their intermembrane space (IMS) several cytotoxic proteins including Omi, SMAC/Diablo, and cytochrome c [4,5,6,7,8]. Following cellular stress and apoptosis induction, the outer mitochondrial membrane (OMM) is permeabilized, leading to their release into the cytosol and subsequent activation of cysteine-aspartic proteases, called caspases [4].

This mitochondrial outer membrane permeabilization (MOMP) is under the tight control of the Bcl-2 family of proteins [9]. Initially discovered within the chromosomal translocations of follicular lymphomas, the Bcl-2 proteins (an acronym for B-cell lymphoma 2 gene) are considered as one of the main MOMP regulators [10,11,12,13,14]. These intracellular proteins possess one or up to four conserved sequences called Bcl-2 homology (BH) domains or motifs [14,15]. As MOMP regulators, they are divided into three groups: multidomain pro-apoptotic Bax-like, which have pore-forming activity and induce MOMP, multidomain anti-apoptotic Bcl-2-like, which bind to Bax-like thus repressing MOMP, and pro-apoptotic BH3-only proteins. Structurally, Bax-like and Bcl-2-like family members are related as they possess four BH motifs (BH1 to 4). The sequence spanning between BH1 to BH3 organizes into the canonical BH3-binding groove where a BH3 motif can bind. In this regard, BH3-only proteins are considered pro-apoptotic as interaction between their BH3 motifs and the BH3-binding groove results in activation of Bax-like or repression of Bcl-2-like proteins, shifting the balance towards MOMP [14,16].

*Bcl2*-related genes are found only in multicellular animals and thus they are referred to as markers of multicellularity, evolutionarily selected to regulate apoptotic cell removal in development and sustain tissue homeostasis in metazoans [17,18]. This was first demonstrated in *C. elegans* in which the Bcl-2 homolog CED-9 was mutated. Loss-of-function of the *ced9* gene resulted in widespread death of embryonic cells [19,20]. Subsequent observations in knockout (KO) mice for *bcl2* homologs solidified their critical role in apoptosis regulation [21,22,23]. However, more recent experiments have demonstrated that Bcl-2 family members are actually multifunctional proteins involved in non-MOMP related processes [24,25,26,27,28]. Indeed, many Bcl-2-related proteins have a C-terminal hydrophobic transmembrane (TM) motif allowing them to be anchored not only to mitochondria but also to the endoplasmic reticulum (ER) [29,30,31,32,33]. At the level of these internal membranes, Bcl-2 family members dynamically control the exchange of Ca^2+^ ions [34,35,36,37].

Ca^2+^ ions are important secondary messengers participating in many cellular functions [38]. Ca^2+^ is able to enhance mitochondrial bioenergetics by promoting the activities of pyruvate dehydrogenase, isocitrate dehydrogenase and α-ketoglutarate dehydrogenase [39]. In contrast, mitochondrial Ca^2+^ is also required for the efficient execution of apoptosis, while Ca^2+^ overload induces the opening of the elusive mitochondrial permeability transition pore (mPTP), leading to necrotic cell death [40,41]. The role of Ca^2+^ in the balance between life and death underlies the need for tight regulation of mitochondrial Ca^2+^ pools. Bcl-2 family of proteins participates in this process through direct interactions with various intracellular Ca^2+^ transporters or channels, which have profound consequences for mitochondrial Ca^2+^ homeostasis and downstream Ca^2+^ signalling pathways. Here, we review the role of Bcl-2 proteins in mitochondrial Ca^2+^ homeostasis and how this regulation orchestrates not only survival/death decisions but also non-apoptotic processes like cell migration.

## 2. Mitochondria-ER Contacts (MERCs): A Signalling Platform for Mitochondrial Ca^2+^ Homeostasis

Mitochondria are dynamic intracellular organelles that can store and exchange with the surrounding environment substantial amounts of Ca^2+^ ions [42]. As mitochondria are encompassed by a double membrane, Ca^2+^ is required to cross both layers in order to reach the matrix. The inner mitochondrial membrane (IMM) mitochondrial Ca^2+^ uniporter (MCU) has a low affinity for Ca^2+^, with a K_d_ of 10 µM [43]. This therefore means that mitochondria are unable to uptake Ca^2+^ directly from the cytosol, but rather require the direct transfer of Ca^2+^ from other stores through membrane contact sites (MCS), in order to be able to maintain their Ca^2+^ pools [44]. While lysosomes [45] can directly transfer Ca^2+^ to mitochondria, the most understood pathway is the transfer of Ca^2+^ from the ER to mitochondria at mitochondria-ER contacts (MERCs) [46]. Mitochondria engage in MCS with the ER forming specialized structures known as MERCs or mitochondria-associated ER membranes (MAMs). Around 20% of the mitochondrial surface is involved in ER contacts, with average inter-organelle distances ranging around 10 to 50 nm [47,48]. MERCs are formed and stabilized by tethering proteins, such as Mitofusin-2 (MFN2) [49] or PDZ domain-containing 8 (PDZD8) [50] in mammals regulating the optimal distance between both organelles. This mitochondria-ER interface is essential for several other processes including the synthesis and exchange of lipids, autophagosome formation and mitochondrial dynamics, thus providing a signalling platform to coordinate cell fate [51,52,53].

**Figure 1:**
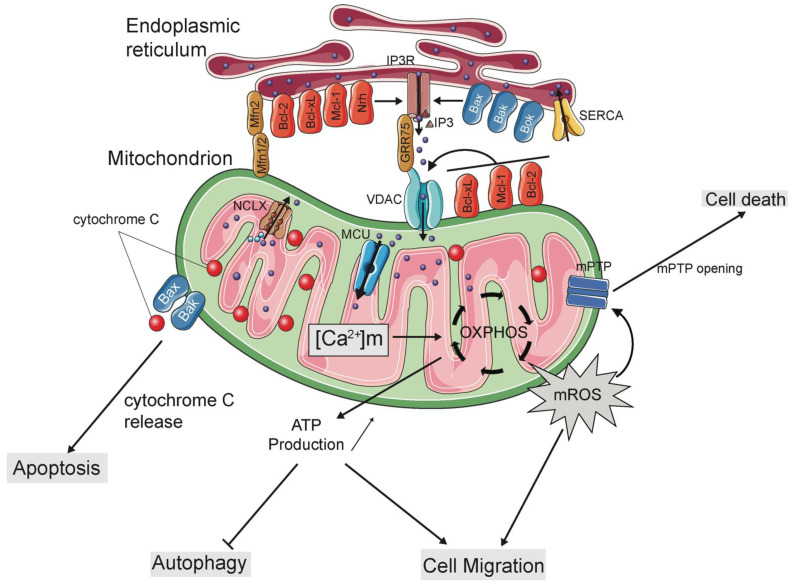
Schematic representation of ER to mitochondria Ca^2+^ regulation by Bcl-2 proteins. Members of the Bcl-2 family including pro- and anti-apoptotic proteins are found at the mitochondria-endoplasmic reticulum contacts (MERCs). At this interface, they control mitochondrial Ca^2+^ trafficking via the interaction with ER- and mitochondria-localized Ca^2+^ channels and transporters, which has an important implication in mitochondrial Ca^2+^-dependent processes. Through mitochondrial Ca^2+^ pools regulation, Bcl-2 proteins control bioenergetics, ATP production and reactive oxygen species (ROS), thus influencing cell fate decisions including apoptosis, cell survival and cell migration. At the ER, the anti-apoptotic proteins Bcl-2, Bcl-xL, Mcl-1 and Nrh interact with IP_3_R to decrease ER-Ca^2+^ release into mitochondria to sustain mitochondrial bioenergetics and to protect from Ca^2+^-induced cell death. At the mitochondria, Bcl-2, Bcl-xL and Mcl-1 interact with VDACs to promote or inhibit mitochondrial Ca^2+^ uptake, depending on cell types and the cellular metabolic state. In contrast, the pro-apoptotic members Bax and Bax can also localize to the ER where they promote ER-Ca^2+^ release and cell death. Recently, ER-localized Bok has been shown to directly regulate MERCs number and to interact with IP_3_R promoting ER-Ca^2+^ release and mitochondrial Ca^2+^ uptake required for cell death.

At the MERCs, a specialized subdomain exists to enable the efficient ER to mitochondria Ca^2+^ transfer. The ER-localized inositol 1,4,5-trisphosphate receptor (IP_3_R) and OMM-localized voltage-dependent anion channel (VDAC) are bridged by the glucose regulated protein-75 (GRP75), forming a tethering complex between both organelles [54,55]. Upon stimulation, the natural ligand IP_3_ binds to IP_3_R leading to opening of the channel and subsequent Ca^2+^ release into cytosol and mitochondria, through VDAC [54] (Figure 1). Ca^2+^ transfer at the MERCs generates a high local concentration of Ca^2+^, called Ca^2+^ microdomains, enabling the MCU complex to uptake Ca^2+^ into the mitochondrial matrix [43]. The formation of this functional Ca^2+^ signalling platform at MERCs organizes all of the appropriate machinery required to efficiently transfer Ca^2+^ from the ER to mitochondria. The high local concentration of Ca^2+^ generated at this interface enables the IMM- and MERCs-localized MCU [56] to allow the entry of Ca^2+^ into the matrix. The spatial organization and coordination of the ER-, OMM- and IMM-localized Ca^2+^ channels/receptors are therefore crucial in order for mitochondria to efficiently uptake Ca^2+^.

Interestingly, biochemical subcellular fractionation studies have shown the presence of the anti-apoptotic proteins Bcl-2 and Bcl-xL at MERCs at steady state [57], with the recruitment of Bcl-2 to this specific interface, mediated by TOM20, being enhanced upon apoptotic stimulations [58]. In addition, microscopy analyses revealed that the apoptosis accelerator Bax is recruited to MERCs during tBid-induced apoptosis [59]. Upon mild stress induced by thapsigargin in Chinese hamster ovary (CHO) cells, Bcl-xL can also translocate specifically to MERCs promoting the increase of mitochondrial Ca^2+^ by regulating IP_3_R-induced ER-Ca^2+^ release and cellular bioenergetics [60].

While the subcellular localization of Bcl-2 proteins at this ER-mitochondria interface allows direct interaction with components of the Ca^2+^ homeostasis machinery, there is little evidence showing that Bcl-2 proteins can directly regulate ER and mitochondria membranes apposition. Recently, it has been shown that the pro-apoptotic member Bok was localized at MERCs where it controls the optimal distance between the two membranes for an efficient ER to mitochondria Ca^2+^ transfer to control cell death [61]. These results are in accordance with recent evidence indicating that overexpression of both Mcl-1 and Bok TMs leads to an increase of MERCs number and cell death [32]. An interaction between Bcl-2 and Bcl-xL with GRP75 was also identified, and it may be plausible that this interaction could regulate MERCs by controlling the IP_3_R-GRP75-VDAC tethering complex [62]. Together, these data suggest that Bcl-2 proteins are not only localized to MERCs but could directly regulate them to sustain efficient ER to mitochondria Ca^2+^ transfer (Figure 1).

In the next sections, we will describe how Bcl-2 proteins regulate Ca^2+^ transients at the ER and mitochondrial interface to promote the uptake of mitochondrial Ca^2+^ required for cell death and the complex process of cell migration.

## 3. Regulation of Mitochondrial Ca^2+^ Uptake Machinery by Bcl-2 Family Proteins

The OMM is highly permeable to ions and low molecular weight molecules, due to the presence of VDACs, whereas the IMM-localized MCU complex enables Ca^2+^ uptake into the matrix [63,64,65]. Three VDAC isoforms are found in vertebrates (VDAC1–3), representing the most abundant proteins of the OMM [66]. They can adopt two conformational stages: an open state, observed at low membrane potential (−10 mV to +10 mV), which is permeable for cations and small anionic metabolites, and a closed state at high mitochondrial membrane potential exhibiting only cation permeability [67]. All three VDAC isoforms are able to transfer Ca^2+^ ions through the OMM, however, functional implications differ. For instance, VDAC1 allows the passage of the low-amplitude apoptotic Ca^2+^ signals following IP_3_R stimulation [68], whereas VDAC2 is involved in transfer of Ca^2+^ from sarcoplasmic reticulum (SR) and in the rhythmicity of cardiomyocytes [69].

Bcl-2 family members are mainly OMM-resident proteins, so they exert a control over mitochondrial Ca^2+^ uptake mainly through the control of VDACs permeability (Figure 1), however this regulation is still a matter of debate. The first evidence for the implication of a Bcl-2 family member in VDAC permeability came from Craig Thompson’s lab in the late 90s. Vander Heiden and collaborators demonstrated that following growth factor deprivation, cells overexpressing Bcl-xL survive by sustaining ATP/ADP exchanges in the mitochondria, suggesting that Bcl-xL maintains VDAC in an open state [36,70]. Supporting this model, the dephosphorylation of the BH3-only protein Bad, which causes its translocation to the OMM, disrupts the interaction between Bcl-xL and VDAC leading to mitochondrial Ca^2+^ overload [71]. However, using liposomes embedded with purified VDAC proteins, Shimizu and colleagues demonstrated that Bcl-xL binds to and inhibits VDAC opening [72]. Interestingly, in this latter experimental system pro-apoptotic Bax and Bak have the opposite effect and lead to VDAC opening [72].

More recently, the team of Chi Li demonstrated that *bclx* KO mouse embryonic fibroblasts (MEFs) uptake less Ca^2+^ into the mitochondria compared to control cells [73]. Notably, mitochondrial Ca^2+^ uptake was restored when KO MEFs were complemented with exogenous Bcl-xL targeted to the mitochondria but not to the ER. It has then been proposed that Bcl-xL promotes mitochondrial Ca^2+^ entry via direct interaction with the VDAC1 and VDAC3 channels [74], and that its N-terminal BH4 motif was required for this interaction and therefore Ca^2+^ regulation [57]. In this respect, a peptide corresponding to the BH4 motif of Bcl-xL reduces agonist-induced mitochondrial Ca^2+^ uptake and protects cells from apoptosis [57,75].

Actually, within the Bcl-2 family, VDACs participate in functional interactions with Bcl-2 and Mcl-1 as well. Several studies have shown that Bcl-2 interacts with VDAC1 N-terminal α-helix, thereby leading to a reduction in mitochondrial Ca^2+^ uptake [76]. This probably also requires the BH4 motif of Bcl-2 because peptides corresponding to this region close VDAC and suppress pro-apoptotic stimuli [77]. In contrast, overexpression of Bcl-2 in neurons and myotubes has opposite effects, leading to an increase of mitochondrial Ca^2+^ [78,79]. Finally, Mcl-1 has also been shown to directly interact with VDACs to promote mitochondrial Ca^2+^ uptake and bioenergetics in a non-small cell carcinoma cell line [80].

The discrepancies regarding mitochondrial Ca^2+^ trafficking highlight the complex interactions between anti-apoptotic Bcl-2 proteins and VDACs. Due to their role in cell survival and death, it can be hypothesized that under physiological conditions, anti-apoptotic Bcl-2 members enhance mitochondrial Ca^2+^ uptake to regulate mitochondrial metabolism and bioenergetics, whereas upon apoptotic stimulations they protect mitochondria from deleterious massive Ca^2+^ overload by interacting with VDACs. This hypothesis is notably supported by findings in the heart of transgenic mice describing that Bcl-2 decreases mitochondrial Ca^2+^ efflux via the Na^+^/Ca^2+^ exchanger, NCLX, to maintain mitochondrial ATP production [81].

## 4. Remote Control of Mitochondrial Ca^2+^ Signalling by ER-Based Bcl-2 Proteins

The ER is the major storage organelle for cellular Ca^2+^. ER-dependent Ca^2+^ release controls basal cytosolic Ca^2+^ levels and mitochondrial Ca^2+^ uptake through the direct transfer of Ca^2+^ ions at MERCs [42,48,82]. At the level of the ER, this occurs via the release of Ca^2+^ through ER-Ca^2+^ channels. IP_3_Rs and ryanodine receptors (RyRs) are the two major families of ER Ca^2+^ channels [83]. In vertebrates, there are three IP_3_R isoforms (IP_3_R1-3), which are often co-expressed in most mammalian cell types. The three isoforms differ in their affinity for the IP_3_ ligand; IP_3_R2 exhibiting the highest sensitivity while IP_3_R3 has the weakest [84]. Interestingly, IP_3_R2 isoform is the most effective in delivering Ca^2+^ to the mitochondria [55]. IP_3_, the natural ligand for IP_3_R, is produced upon G-protein coupled receptor (GPCR) activation by ligands such as histamine or ATP at the plasma membrane. GPCR activation leads to hydrolysis of phosphatidyl inositol-4,5-bisphosphate (PIP_2_) by phospholipase C resulting in the production of IP_3_. IP_3_ then diffuses through the cell and binds to the IP_3_-binding domain of IP_3_R oligomers resulting in the opening of the Ca^2+^ channel, which subsequently allows Ca^2+^ flux into the cytosol and mitochondria [85,86].

Actually, many anti-apoptotic Bcl-2 proteins including Bcl-2, Bcl-xL, Mcl-1 and Nrh, possess dual mitochondrial and ER localizations and are able to interact with IP_3_R [34,35,87] (Figure 1). Although these interactions regulate IP_3_R-dependent Ca^2+^ release, the binding sites and the functional consequences of this regulation are different. For instance, Bcl-2 is able to bind to the central modulatory and transducing domain II (MTD II) of IP_3_R, which requires the N-terminal BH4 motif of Bcl-2 [34,88]. This interaction lowers Ca^2+^ release from the ER and inhibits the transfer of toxic Ca^2+^ insults to the mitochondria. Conversely, Bcl-xL interacts with IP_3_R through its BH3-binding groove [89]. Indeed, Yang and colleagues identified two new BH3-like helices in the IP_3_R C-terminus that are able to bind to Bcl-xL with high affinity. This interaction leads to IP_3_R opening and subsequent ER-Ca^2+^ release. Interestingly, the mode of action of Bcl-xL appears to be concentration-dependent because increasing Bcl-xL levels lead to a secondary IP_3_R inhibition, which occurs through the binding of Bcl-xL at the Bcl-2 interaction site in the MTDII domain [89]. Thus, the regulation of IP_3_R by Bcl-xL seems to be biphasic. At low levels, ER-based Bcl-xL favors the release of Ca^2+^ ions from IP_3_R and transfer to the mitochondria thus enhancing mitochondrial bioenergetics by activating the Ca^2+^-dependent dehydrogenases of the Krebs cycle. In contrast, at high protein concentration levels, Bcl-xL inhibits IP_3_R-dependent Ca^2+^ release and subsequent apoptosis initiation [89,90]. Of note, as described previously, the BH4 motif of Bcl-xL does not interact with IP_3_R but preferentially binds to VDAC1, controlling its permeability [57]. The difference between Bcl-2 and Bcl-xL BH4 motifs can be explained by subtle differences in their respective amino acid compositions. Indeed, in the BH4 motif of Bcl-2 a lysine residue at position 17 (Lys17) is critical for its interaction with IP_3_R. The corresponding residue in Bcl-xL is an aspartate at position 11 (Asp11). Mutating Lys17 into Asp in Bcl-2 leads to complete loss of IP_3_R binding capacity, whereas changing of Asp11 into Lys in BH4 of Bcl-xL converts Bcl-xL into an IP_3_R binder and inhibitor [57].

Mcl-1 is another IP_3_R interactor shown to control mitochondrial Ca^2+^ uptake. Actually, Mcl-1 and Bcl-xL seem to behave in a similar manner. Both proteins bind with comparable affinities to the C-terminus of all three IP_3_R isoforms suggesting that Mcl-1, like Bcl-xL, requires its BH3-binding groove to interact with IP_3_R channels [91]. In addition, the BH4 motif of Mcl-1 has a pronounced tropism for the OMM, where it inhibits mitochondrial Ca^2+^ signalling [92].

An outsider of this Bcl-2-IP_3_R interaction group is the Bcl-2 homolog Nrh (also referred to as Bcl-B or BCL2L10). In breast cancer (BC) cells, Nrh is exclusively found at the ER where it is able to interact with the N-terminal IP_3_ binding domain of the IP_3_R1 via its BH4 motif [87]. This interaction prevents IP_3_R1 opening, which in turn dampens the unfolded protein response (UPR). Actually, the UPR is an adaptive reaction that prevents the accumulation of misfolded proteins in the ER lumen to maintain cell viability. If stressful conditions persist, the UPR can prime cells for cell death through the activation of the BH3-only protein Bim [93,94]. The UPR is often suppressed in tumor cells in order to promote protein synthesis and cell survival. In this regard, Nrh expression in BC cells inhibits the UPR and induces drug resistance, whereas Nrh silencing makes BC cells more sensitive to drugs currently used in chemotherapy [87]. Interestingly, at MERCs, Nrh and IRBIT, another IP_3_ binding domain protein, exert an additive inhibitory effect over IP_3_R at resting states [95]. However, upon apoptotic stress, IRBIT is dephosphorylated, thus inhibiting Nrh and leading to Ca^2+^ accumulation in the mitochondria and subsequent apoptosis [95].

Finally, Bcl-2 proteins have also been proposed to interact with other ER-Ca^2+^ channels [96]. Both Bcl-2 and Bcl-xL can interact with the ryanodine receptor (Ryr) via their BH4 domains and decrease their activity [97,98]. Indeed, overexpression of Bcl-xL inhibits caffeine-induced Ryr-dependent Ca^2+^ release into the mitochondria [98]. Together, by direct interaction with ER-Ca^2+^ channels, Bcl-2 proteins tightly control ER to mitochondria Ca^2+^ transfer required for cell fate decisions.

## 5. Bridging the Gap between Mitochondria and ER during Cell Death and Survival

Mitochondrial Ca^2+^ plays a pivotal role in the balance between cell survival and cell death events [99]. While a minimal amount of Ca^2+^ is required to maintain mitochondrial bioenergetics and metabolism, larger and toxic mitochondrial Ca^2+^ levels have been proposed to facilitate apoptosis [100] and to trigger mPTP opening [41]. As already described, the anti-apoptotic Bcl-2 proteins are key executioners regarding the control of mitochondrial Ca^2+^ homeostasis as well as the cell death and survival balance. During apoptosis, the number of mitochondria-ER contact sites increases [59,101], fostering mitochondrial Ca^2+^ uptake [59,95], which has been associated with IMM remodelling and OPA1-dependent cristae reorganization, thus facilitating cytochrome c release and apoptosis [59]. Mitochondrial Ca^2+^ overload may also lead to IMM cardiolipin oxidation, increasing ROS production and mPTP opening [102]. Due to their roles in ER to mitochondria Ca^2+^ fluxes described above, anti-apoptotic Bcl-2 proteins have been widely shown to inhibit apoptosis by decreasing ER-induced Ca^2+^ release or decreasing VDAC1-dependent Ca^2+^ uptake [103] (Figure 1). In recent years, different peptides derived from their BH4 domain have been developed and their effects have been characterized in different cancer cell models [104]. Such peptides are able to disrupt the interactions between IP_3_R and several Bcl-2 proteins and impact on the apoptotic Ca^2+^ signals transfer to the mitochondria. For instance, a BH4-domain-targeting peptide of Bcl-2, called Bcl-2/IP_3_ receptor disrupter-2 (BIRD-2), has been shown to have cell death-inducing effects in different cancer cell lines [34,105,106,107,108]. Interestingly, such cell death has been shown to depend on ER-induced mitochondrial Ca^2+^ overload and caspase activation [109].

While the role of the pro-apoptotic proteins in basal mitochondrial Ca^2+^ homeostasis has been less described, there is evidence supporting their contribution to the Ca^2+^-dependent apoptotic process (Figure 1). Bok is the only multidomain pro-apoptotic member which has been shown to interact with the IP_3_R coupling domain of both IP_3_R1 and IP_3_R2 via its BH4 domain [110,111]. This interaction has been initially reported to protect both IP_3_Rs and unbound Bok from proteolysis and proteasomal-dependent degradation, respectively, and to control mitochondrial morphology [112]; however, no ER or mitochondrial Ca^2+^ defects were observed in these KO cell lines. Interestingly, a study has recently shown that KO of Bok resulted in a deregulation of intracellular Ca^2+^ signalling [61]. Indeed, these Bok KO MEFs harbored a reduction of Ca^2+^ transfer from ER to mitochondria and of apoptosis [61]. This study also showed that Bok-KO induces a decrease of MERCs number observed by microscopy, and a mislocalization and decrease of MERCs-resident proteins [61], suggesting that Bok can directly control MERCs to maintain mitochondrial Ca^2+^ pools and sustain cell viability. Rescue experiments with a Bok mutant unable to interact with IP_3_R was shown to rescue the MERCs defect but not the mitochondrial Ca^2+^ phenotype [61]. Interestingly, restoring MERCs by an artificial tether, was insufficient to recuse the Ca^2+^ defects induced by Bok loss [61]. These data suggest a specific and mutually exclusive role of Bok in controlling IP_3_R-mediated Ca^2+^ release and MERCs number.

Although no direct interaction with ER-localized Ca^2+^ channels/receptors have been reported, Bax and Bak can also localize to the ER where they control Ca^2+^-dependent apoptosis [113,114,115]. Indeed, overexpression of Bax and Bak leads to an increase of ER-Ca^2+^ release and mitochondrial Ca^2+^ levels accompanied by cytochrome c release and cell death [113], suggesting that Bax/Bak at the ER can control ER to mitochondria Ca^2+^ fluxes. In addition, Bax/Bak DKO MEFs have reduced ER-Ca^2+^ content, leading to decreased mitochondrial Ca^2+^ uptake and apoptosis upon ER-Ca^2+^ stimulation [114]. Importantly, re-expression of SERCA or ER-targeted Bax/Bak was able to restore ER-Ca^2+^ content and efficient apoptosis, indicating that Bax and Bak directly control ER Ca^2+^ concentration [114]. Mechanistically, it has been proposed that this increased ER Ca^2+^ leak was associated to an increase of Bcl-2-IP_3_R1 interaction and protein kinase A-dependent IP_3_R1 phosphorylation in Bax and Bak DKO cells [116]. Other studies have confirmed the contribution of Bax and Bak regarding ER-induced Ca^2+^ release and cell death regulation following different cellular stresses [115,117]. Alternatively, reports have shown that Bax and Bak are able to permeabilize the ER membrane leading to the release of the ER lumen contents to the cytosol [118,119]. Indeed, the oligomerization of Bax and Bak on the ER membrane could lead to the formation of pores, similar to mitochondrial [120,121] and peroxisomal [122] pore formations, which could potentially allow the passage of Ca^2+^ in the cytosol during apoptosis. Finally, BH3 only proteins [123,124], including Bik [125], can also control Bax/Bak-dependent ER-Ca^2+^ release to enhance mitochondrial Ca^2+^ uptake and cell death. In hyperplastic cells, not only Bik disrupts the Bcl-2-IP_3_R complex to promote ER-Ca^2+^ release, but it can also activate and translocate Bak to the ER to form a complex with DAPK1 leading to an increase of MERCs and mitochondrial Ca^2+^ uptake, subsequently leading to cell death [126].

The complex regulation of Ca^2+^ by Bcl-2 proteins reflects the critical and opposing functions of Ca^2+^ about life and death decisions. Therefore, several modes of regulation must exist to tightly control mitochondrial Ca^2+^ levels, depending on environmental conditions.

## 6. Role of Bcl-2 Family Proteins in Ca^2+^-Dependent Cell Migration

Intracellular Ca^2+^ dynamics regulates many cellular processes including cytoskeleton remodelling and cell migration [37]. Most of these regulations occur by modifying the cytosolic Ca^2+^ signals, which has been reviewed extensively elsewhere [127,128]. The significance of Bcl-2 family proteins in cell migration and invasion during embryonic development and cancer progression, however, has only recently emerged.

Actually, the first evidence came from experiments conducted in the zebrafish model. In this vertebrate, a highly divergent Bcl-2 homolog, called Bcl-wav (acronym for Bcl-2 homolog found in water-living anamniote vertebrates) was identified [28]. Bcl-wav is a mitochondrial resident pro-apoptotic Bcl-2 homolog, the knockdown of which affects convergence and extension (C&E) movements during zebrafish embryogenesis [28]. C&E movements are critical for the establishment of the anterior-posterior and dorsoventral embryonic axes. Bcl-wav orchestrates these morphogenic movements through the control of intracellular Ca^2+^ trafficking. Indeed, *bclwav* knockdown was correlated with a decrease in mitochondrial Ca^2+^ levels and concomitant increase of cytosolic Ca^2+^ levels [28]. At the level of the mitochondria, Bcl-wav interacts with VDAC1 via its BH4 motif and enhances mitochondrial Ca^2+^ uptake thus controlling the kinetics of actin polymerization and blastomeres migration. Interestingly, C&E movements seem to be strongly depended on mitochondrial Ca^2+^ uptake since knockdown of *mcu* resulted in a similar phenotype [28].

The importance of the MCU-dependent Ca^2+^ transport was further emphasized in the motility of cancer cells [129,130]. Indeed, *mcu*-silencing in highly invasive triple-negative breast cancer (TNBC) cell lines resulted in altered F-actin cytoskeleton dynamics, cell polarization loss and impairment of the focal adhesion proteins dynamics [129]. These processes are mediated by the reduction of Ca^2+^-dependent Calpain and Rho-GTPases activities [129]. In addition, Tosatto and collaborators showed that the knockdown of *mcu* also resulted in decreased cell motility and invasiveness as well as reduction of tumor growth [130]. However, they linked this phenotype to mitochondrial ROS (mtROS) production and downregulation of hypoxia-inducible factor-1α (HIF-1α) [130]. This suggests that mitochondrial Ca^2+^ uptake could probably control multiple downstream signalling pathways. High mtROS production is detrimental for cell survival, however, in cancer cells sub-lethal mtROS levels promote cell proliferation, migration and invasion [131,132]. In this respect, several studies have demonstrated that Bcl-2 family members control cancer cell motility via mtROS production, independently of their role in apoptosis [80,133,134]. For instance, Mcl-1 was proposed to promote migration in non-small cell lung carcinoma though its interaction with VDAC1 and 3 and its capacity to control mitochondrial Ca^2+^ homeostasis [80]. Indeed, *mcl1*-silencing or treatment with peptides that suppress VDAC-based Ca^2+^ uptake led to reduced mtROS generation. Bcl-xL and Bcl-2 were also shown to act as accelerators of cell motility, invasiveness and metastasis spreading. As it is the case for Mcl-1, mitochondrion-localized Bcl-xL, but not ER-based Bcl-xL, contributes to cell migration through the generation of reactive mtROS [133]. At the level of the mitochondria, Bcl-xL binds to VDAC1 via its BH4 motif thus promoting Ca^2+^ entry and mtROS production. Interestingly, one study linked this regulation with the effect of metalloprotease-processed CD95L (cl-CD95L) on TNBC accelerated metastatic dissemination and poor patient prognosis [134]. Actually, CD95-mediates Ca^2+^ release from the ER to mitochondria at MERCs. In this particular case, mitochondria-targeted Bcl-xL and ER-targeted Bcl-2 were proposed to increase Ca^2+^ transfer between the ER and the mitochondria, thus accelerating ATP production and mtROS generation [134]. Interestingly in this case, the use of BH3-mimetics was sufficient to decrease cell migration suggesting that these molecules may be useful not only to kill tumor cells but also to prevent metastatic dissemination [134].

## 7. Conclusions

The role of Bcl-2 family of proteins in the initiation of apoptosis has been well studied, which has led to our current understanding of how cells integrate stress signals at the level of the mitochondria, leading to initiation of the death program. The role of Ca^2+^ in mediating cell death decisions has also been emphasized, but recent evidence support additional functions for mitochondrial Ca^2+^ on top of mitochondrial bioenergetics and cell death. With their capacity to be localized at the mitochondria-ER interface and to interact with keys channels or receptors on both ER and mitochondrial membranes, Bcl-2 proteins have emerged as key regulators of intracellular and mitochondrial Ca^2+^ homeostasis, and subsequently to several other processes such as cell migration. Due to this connection, numerous studies are currently directly targeting Bcl-2-IP_3_R or Bcl-2-VDAC interactions to modulate Ca^2+^ signalling and to control cell fate in different types of cancer cell models. Together, future studies identifying precisely how mitochondrial Ca^2+^ is regulated by Bcl-2 proteins may identify new strategies for therapeutic intervention.

## References

[1] Elmore S. (2007). Apoptosis: A review of programmed cell death. Toxicol. Pathol..

[2] Letai A. (2017). Apoptosis and Cancer. Annu. Rev. Cancer Biol..

[3] Mattson M.P. (2000). Apoptosis in neurodegenerative disorders. Nat. Rev. Mol. Cell Biol..

[4] Liu X., Kim C.N., Yang J., Jemmerson R., Wang X. (1996). Induction of Apoptotic Program in Cell-Free Extracts: Requirement for dATP and Cytochrome c. Cell.

[5] Li P., Nijhawan D., Budihardjo I., Srinivasula S.M., Ahmad M., Alnemri E.S., Wang X. (1997). Cytochrome c and dATP-Dependent Formation of Apaf-1/Caspase-9 Complex Initiates an Apoptotic Protease Cascade. Cell.

[6] Verhagen A.M., Ekert P.G., Pakusch M., Silke J., Connolly L.M.E., Reid G., Moritz R.L., Simpson R.J., Vaux D.L. (2000). Identification of DIABLO, a Mammalian Protein that Promotes Apoptosis by Binding to and Antagonizing IAP Proteins. Cell.

[7] Du C., Fang M., Li Y., Li L., Wang X. (2000). Smac, a Mitochondrial Protein that Promotes Cytochrome c–Dependent Caspase Activation by Eliminating IAP Inhibition. Cell.

[8] Suzuki Y., Imai Y., Nakayama H., Takahashi K., Takio K., Takahashi R. (2001). A Serine Protease, HtrA2, Is Released from the Mitochondria and Interacts with XIAP, Inducing Cell Death. Mol. Cell.

[9] Singh R., Letai A., Sarosiek K. (2019). Regulation of apoptosis in health and disease: The balancing act of BCL-2 family proteins. Nat. Rev. Mol. Cell Biol..

[10] Tsujimoto Y., Cossman J., Jaffe E., Croce C.M. (1985). Involvement of the bcl-2 gene in human follicular lymphoma. Science.

[11] Tsujimoto Y., Finger L.R., Yunis J., Nowell P.C., Croce C.M. (1984). Cloning of the chromosome breakpoint of neoplastic B cells with the t(14;18) chromosome translocation. Science.

[12] Tsujimoto Y., Croce C.M. (1986). Analysis of the structure, transcripts, and protein products of bcl-2, the gene involved in human follicular lymphoma. Proc. Natl. Acad. Sci. USA.

[13] Delbridge A.R.D., Grabow S., Strasser A., Vaux D.L. (2016). Thirty years of BCL-2: Translating cell death discoveries into novel cancer therapies. Nat. Rev. Cancer.

[14] Youle R.J., Strasser A. (2008). The BCL-2 protein family: Opposing activities that mediate cell death. Nat. Rev. Mol. Cell Biol..

[15] Banjara S., Suraweera C.D., Hinds M.G., Kvansakul M. (2020). The Bcl-2 Family: Ancient Origins, Conserved Structures, and Divergent Mechanisms. Biomolecules.

[16] Kale J., Osterlund E.J., Andrews D.W. (2018). BCL-2 family proteins: Changing partners in the dance towards death. Cell Death Differ..

[17] Strasser A., Vaux D.L. (2017). Viewing BCL2 and cell death control from an evolutionary perspective. Cell Death Differ..

[18] Green D.R., Fitzgerald P. (2016). Just So Stories about the Evolution of Apoptosis. Curr. Biol..

[19] Hengartner M.O., Ellis R., Horvitz R. (1992). Caenorhabditis elegans gene ced-9 protects cells from programmed cell death. Nat. Cell Biol..

[20] Hengartner M.O., Horvitz H.C. (1994). elegans cell survival gene ced-9 encodes a functional homolog of the mammalian proto-oncogene bcl-2. Cell.

[21] Veis D.J., Sorenson C.M., Shutter J.R., Korsmeyer S.J. (1993). Bcl-2-deficient mice demonstrate fulminant lymphoid apoptosis, polycystic kidneys, and hypopigmented hair. Cell.

[22] Knudson C.M., Tung K.S., Tourtellotte W.G., Brown G.A., Korsmeyer S.J. (1995). Bax-deficient mice with lymphoid hyperplasia and male germ cell death. Science.

[23] Rinkenberger J.L., Horning S., Klocke B., Roth K., Korsmeyer S.J. (2000). Mcl-1 deficiency results in peri-implantation embryonic lethality. Genome Res..

[24] Wang X., Belguise K., Kersual N., Kirsch K.H., Mineva N.D., Galtier F., Chalbos D., Sonenshein G.E. (2007). Oestrogen signalling inhibits invasive phenotype by repressing RelB and its target BCL2. Nat. Cell Biol..

[25] Li H., Alavian K.N., Lazrove E., Mehta N., Jones A., Zhang P., Licznerski P., Graham M., Uo T., Guo J. (2013). A Bcl-Xl-Drp1 Complex Regulates Synaptic Vesicle Membrane Dynamics During Endocytosis. Nat Cell Biol.

[26] Wu X., Zhang L.-S., Toombs J., Kuo Y.-C., Piazza J.T., Tuladhar R., Barrett Q., Fan C.-W., Zhang X., Walensky L.D. (2017). Extra-mitochondrial prosurvival BCL-2 proteins regulate gene transcription by inhibiting the SUFU tumour suppressor. Nat. Cell Biol..

[27] Popgeorgiev N., Bonneau B., Ferri K.F., Prudent J., Thibaut J., Gillet G. (2011). The Apoptotic Regulator Nrz Controls Cytoskeletal Dynamics via the Regulation of Ca2+ Trafficking in the Zebrafish Blastula. Dev. Cell.

[28] Prudent J., Popgeorgiev N., Bonneau B., Thibaut J., Gadet R., Lopez J., Gonzalo P., Rimokh R., Manon S., Houart C. (2013). Bcl-wav and the mitochondrial calcium uniporter drive gastrula morphogenesis in zebrafish. Nat. Commun..

[29] Krajewski S., Tanaka S., Takayama S., Schibler M.J., Fenton W., Reed J.C. (1993). Investigation of the subcellular distribution of the bcl-2 oncoprotein: Residence in the nuclear envelope, endoplasmic reticulum, and outer mitochondrial membranes. Cancer Res..

[30] Yang T., Kozopas K.M., Craig R.W. (1995). The intracellular distribution and pattern of expression of Mcl-1 overlap with, but are not identical to, those of Bcl-2. J. Cell Biol..

[31] Kaufmann T., Schlipf S., Sanz J., Neubert K., Stein R., Borner C. (2003). Characterization of the signal that directs Bcl-xL, but not Bcl-2, to the mitochondrial outer membrane. J. Cell Biol..

[32] Lucendo E., Sancho M., Lolicato F., Javanainen M., Kulig W., Leiva D., Duart G., Andreu-Fernández V., Mingarro I., Orzáez M. (2020). Mcl-1 and Bok transmembrane domains: Unexpected players in the modulation of apoptosis. Proc. Natl. Acad. Sci. USA.

[33] Popgeorgiev N., Jabbour L., Gillet G. (2018). Subcellular Localization and Dynamics of the Bcl-2 Family of Proteins. Front. Cell Dev. Biol..

[34] Rong Y.-P., Aromolaran A.S., Bultynck G., Zhong F., Li X., McColl K., Matsuyama S., Herlitze S., Roderick H.L., Bootman M.D. (2008). Targeting Bcl-2-IP3 Receptor Interaction to Reverse Bcl-2’s Inhibition of Apoptotic Calcium Signals. Mol. Cell.

[35] White C., Li C., Yang J., Petrenko N.B., Madesh M., Thompson C.B., Foskett J.K. (2005). The Endoplasmic Reticulum Gateway to Apoptosis by Bcl-X(L) Modulation of the Insp3r. Nat. Cell Biol..

[36] Vander Heiden M.G., Li X.X., Gottleib E., Hill R.B., Thompson C.B., Colombini M. (2001). Bcl-Xl Promotes the Open Configuration of the Voltage-Dependent Anion Channel and Metabolite Passage through the Outer Mitochondrial Membrane. J. Biol. Chem..

[37] Bonneau B., Prudent J., Popgeorgiev N., Gillet G. (2013). Non-apoptotic roles of Bcl-2 family: The calcium connection. Biochim. Biophys. Acta BBA Bioenerg..

[38] Berridge M.J., Lipp P., Bootman M.D. (2000). The versatility and universality of calcium signalling. Nat. Rev. Mol. Cell Biol..

[39] Denton R.M. (2009). Regulation of mitochondrial dehydrogenases by calcium ions. Biochim. Biophys. Acta BBA Bioenerg..

[40] Bernardi P., von Stockum S. (2012). The Permeability Transition Pore as a Ca(2+) Release Channel: New Answers to an Old Question. Cell Calcium.

[41] Bauer T.M., Murphy E. (2020). Role of Mitochondrial Calcium and the Permeability Transition Pore in Regulating Cell Death. Circ. Res..

[42] Raffaello A., Mammucari C., Gherardi G., Rizzuto R. (2016). Calcium at the Center of Cell Signaling: Interplay between Endoplasmic Reticulum, Mitochondria, and Lysosomes. Trends Biochem. Sci..

[43] Kamer K.J., Mootha V.K. (2015). The molecular era of the mitochondrial calcium uniporter. Nat. Rev. Mol. Cell Biol..

[44] Rizzuto R., Pinton P., Carrington W., Fay F.S., Fogarty K.E., Lifshitz L.M., Tuft R.A., Pozzan T. (1998). Close Contacts with the Endoplasmic Reticulum as Determinants of Mitochondrial Ca2+ Responses. Science.

[45] Peng W., Wong Y.C., Krainc D. (2020). Mitochondria-lysosome contacts regulate mitochondrial Ca2+dynamics via lysosomal TRPML1. Proc. Natl. Acad. Sci. USA.

[46] Vallese F., Barazzuol L., Maso L., Brini M., Calì T. (2019). ER-Mitochondria Calcium Transfer, Organelle Contacts and Neurodegenerative Diseases. Adv. Exp. Med. Biol..

[47] Giacomello M., Pyakurel A., Glytsou C., Scorrano L. (2020). The cell biology of mitochondrial membrane dynamics. Nat. Rev. Mol. Cell Biol..

[48] Csordás G., Várnai P., Golenár T., Roy S., Purkins G., Schneider T.G., Balla T., Hajnóczky G. (2010). Imaging Interorganelle Contacts and Local Calcium Dynamics at the ER-Mitochondrial Interface. Mol. Cell.

[49] De Brito O.M., Scorrano L. (2008). Mitofusin 2 Tethers Endoplasmic Reticulum to Mitochondria. Nature.

[50] Hirabayashi Y., Kwon S.-K., Paek H., Pernice W.M., Paul M.A., Lee J., Erfani P., Raczkowski A., Petrey D.S., Pon L.A. (2017). ER-mitochondria tethering by PDZD8 regulates Ca2+dynamics in mammalian neurons. Science.

[51] Rowland A.A., Voeltz G.K. (2012). Endoplasmic reticulum–mitochondria contacts: Function of the junction. Nat. Rev. Mol. Cell Biol..

[52] Prudent J., McBride H.M. (2017). The mitochondria–endoplasmic reticulum contact sites: A signalling platform for cell death. Curr. Opin. Cell Biol..

[53] Tilokani L., Nagashima S., Paupe V., Prudent J. (2018). Mitochondrial dynamics: Overview of molecular mechanisms. Essays Biochem..

[54] Szabadkai G., Bianchi K., Várnai P., De Stefani D., Wieckowski M.R., Cavagna D., Nagy A.I., Balla T., Rizzuto R. (2006). Chaperone-mediated coupling of endoplasmic reticulum and mitochondrial Ca2+ channels. J. Cell Biol..

[55] Bartok A., Weaver D., Golenár T., Nichtova Z., Katona M., Bánsághi S., Alzayady K.J., Thomas V.K., Ando H., Mikoshiba K. (2019). IP3 receptor isoforms differently regulate ER-mitochondrial contacts and local calcium transfer. Nat. Commun..

[56] De La Fuente S., Fernandez-Sanz C., Vail C., Agra E.J., Holmstrom K., Sun J., Mishra J., Williams D., Finkel T., Murphy E. (2016). Strategic Positioning and Biased Activity of the Mitochondrial Calcium Uniporter in Cardiac Muscle. J. Biol. Chem..

[57] Monaco G., Decrock E., Arbel N., van Vliet A.R., La Rovere R.M., De Smedt H., Parys J.B., Agostinis P., Leybaert L., Shoshan-Barmatz V. (2015). The BH4 Domain of Anti-apoptotic Bcl-XL, but Not That of the Related Bcl-2, Limits the Voltage-dependent Anion Channel 1 (VDAC1)-mediated Transfer of Pro-apoptotic Ca2+ Signals to Mitochondria. J. Biol. Chem..

[58] Lalier L., Mignard V., Joalland M.-P., Lanoé D., Cartron P.-F., Manon S., Vallette F.M. (2021). TOM20-mediated transfer of Bcl2 from ER to MAM and mitochondria upon induction of apoptosis. Cell Death Dis..

[59] Prudent J., Zunino R., Sugiura A., Mattie S., Shore G.C., McBride H.M. (2015). MAPL SUMOylation of Drp1 Stabilizes an ER/Mitochondrial Platform Required for Cell Death. Mol. Cell.

[60] Williams A., Hayashi T., Wolozny D., Yin B., Su T.C., Betenbaugh M.J., Su T.P. (2016). The Non-Apoptotic Action of Bcl-Xl: Regulating Ca(2+) Signaling and Bioenergetics at the Er-Mitochondrion Interface. J. Bioenerg. Biomembr..

[61] Carpio M.A., Means R.E., Brill A.L., Sainz A., Ehrlich B.E., Katz S.G. (2021). BOK controls apoptosis by Ca2+ transfer through ER-mitochondrial contact sites. Cell Rep..

[62] Saxena N., Katiyar S.P., Liu Y., Grover A., Gao R., Sundar D., Kaul S.C., Wadhwa R. (2013). Molecular Interactions of Bcl-2 and Bcl-Xl with Mortalin: Identification and Functional Characterization. Biosci. Rep..

[63] Becker T., Wagner R. (2018). Mitochondrial Outer Membrane Channels: Emerging Diversity in Transport Processes. BioEssays.

[64] Perocchi F., Gohil V.M., Girgis H.S., Bao X.R., McCombs J.E., Palmer A.E., Mootha V.K. (2010). MICU1 encodes a mitochondrial EF hand protein required for Ca2+ uptake. Nat. Cell Biol..

[65] Baughman J.M., Perocchi F., Girgis H.S., Plovanich M., Belcher-Timme C.A., Sancak Y., Bao X.R., Strittmatter L.A., Goldberger O., Bogorad R.L. (2011). Integrative genomics identifies MCU as an essential component of the mitochondrial calcium uniporter. Nat. Cell Biol..

[66] Raghavan A., Sheiko T., Graham B.H., Craigen W.J. (2012). Voltage-dependant anion channels: Novel insights into isoform function through genetic models. Biochim. Biophys. Acta BBA Biomembr..

[67] Tan W., Colombini M. (2007). VDAC closure increases calcium ion flux. Biochim. Biophys. Acta BBA Biomembr..

[68] De Stefani D., Bononi A., Romagnoli A., Messina A., De Pinto V., Pinton P., Rizzuto R. (2012). Vdac1 Selectively Transfers Apoptotic Ca2+ Signals to Mitochondria. Cell Death Differ..

[69] Shimizu H., Schredelseker J., Huang J., Lu K., Naghdi S., Lu F., Franklin S., Fiji H.D., Wang K., Zhu H. (2015). Mitochondrial Ca2+ uptake by the voltage-dependent anion channel 2 regulates cardiac rhythmicity. eLife.

[70] Vander Heiden M.G., Chandel N.S., Schumacker P.T., Thompson C.B. (1999). Bcl-Xl Prevents Cell Death Following Growth Factor Withdrawal by Facilitating Mitochondrial Atp/Adp Exchange. Mol. Cell.

[71] Roy S.S., Madesh M., Davies E., Antonsson B., Danial N., Hajnóczky G. (2009). Bad Targets the Permeability Transition Pore Independent of Bax or Bak to Switch between Ca2+-Dependent Cell Survival and Death. Mol. Cell.

[72] Shimizu S., Narita M., Tsujimoto Y. (1999). Bcl-2 family proteins regulate the release of apoptogenic cytochrome c by the mitochondrial channel VDAC. Nat. Cell Biol..

[73] Eno C.O., Eckenrode E.F., Olberding K.E., Zhao G., White C., Li C. (2012). Distinct Roles of Mitochondria- and Er-Localized Bcl-Xl in Apoptosis Resistance and Ca2+ Homeostasis. Mol. Biol. Cell.

[74] Huang H., Hu X., Eno C.O., Zhao G., Li C., White C. (2013). An Interaction between Bcl-Xl and the Voltage-Dependent Anion Channel (Vdac) Promotes Mitochondrial Ca2+ Uptake. J. Biol. Chem..

[75] Arbel N., Ben-Hail D., Shoshan-Barmatz V. (2012). Mediation of the Antiapoptotic Activity of Bcl-Xl Protein Upon Interaction with Vdac1 Protein. J. Biol. Chem..

[76] Abu-Hamad S., Arbel N., Calo D., Arzoine L., Israelson A., Keinan N., Ben-Romano R., Friedman O., Shoshan-Barmatz V. (2009). The VDAC1 N-terminus is essential both for apoptosis and the protective effect of anti-apoptotic proteins. J. Cell Sci..

[77] Shimizu S., Konishi A., Kodama T., Tsujimoto Y. (2000). BH4 domain of antiapoptotic Bcl-2 family members closes voltage-dependent anion channel and inhibits apoptotic mitochondrial changes and cell death. Proc. Natl. Acad. Sci. USA.

[78] Jiao J., Huang X., Feit-Leithman R.A., Neve R.L., Snider W., Dartt D.A., Chen D.F. (2005). Bcl-2 enhances Ca2+ signaling to support the intrinsic regenerative capacity of CNS axons. EMBO J..

[79] Basset O., Boittin F.-X., Cognard C., Constantin B., Ruegg U.T. (2006). Bcl-2 overexpression prevents calcium overload and subsequent apoptosis in dystrophic myotubes. Biochem. J..

[80] Huang H., Shah K.H.A., Bradbury N., Li C., White C.M. (2014). Mcl-1 promotes lung cancer cell migration by directly interacting with VDAC to increase mitochondrial Ca2+ uptake and reactive oxygen species generation. Cell Death Dis..

[81] Zhu L., Yu Y., Chua B.H., Ho Y.-S., Kuo T.H. (2001). Regulation of Sodium–Calcium Exchange and Mitochondrial Energetics by Bcl-2 in the Heart of Transgenic Mice. J. Mol. Cell. Cardiol..

[82] Csordás G., Weaver D., Hajnóczky G. (2018). Endoplasmic Reticulum–Mitochondrial Contactology: Structure and Signaling Functions. Trends Cell Biol..

[83] Seo M.-D., Enomoto M., Ishiyama N., Stathopulos P.B., Ikura M. (2015). Structural insights into endoplasmic reticulum stored calcium regulation by inositol 1,4,5-trisphosphate and ryanodine receptors. Biochim. Biophys. Acta BBA Bioenerg..

[84] Newton C.L.A., Mignery G., Südhof T.C. (1994). Co-expression in vertebrate tissues and cell lines of multiple inositol 1,4,5-trisphosphate (InsP3) receptors with distinct affinities for InsP3. J. Biol. Chem..

[85] Taylor C.W., Tovey S.C. (2010). IP3 Receptors: Toward Understanding Their Activation. Cold Spring Harb. Perspect. Biol..

[86] Ivanova H., Vervliet T., Missiaen L., Parys J.B., De Smedt H., Bultynck G. (2014). Inositol 1,4,5-trisphosphate receptor-isoform diversity in cell death and survival. Biochim. Biophys. Acta BBA Bioenerg..

[87] Nougarède A., Popgeorgiev N., Kassem L., Omarjee S., Borel S., Mikaelian I., Lopez J., Gadet R., Marcillat O., Treilleux I. (2018). Breast Cancer Targeting through Inhibition of the Endoplasmic Reticulum-Based Apoptosis Regulator Nrh/BCL2L10. Cancer Res..

[88] Rong Y.-P., Barr P., Yee V.C., Distelhorst C.W. (2009). Targeting Bcl-2 based on the interaction of its BH4 domain with the inositol 1,4,5-trisphosphate receptor. Biochim. Biophys. Acta BBA Bioenerg..

[89] Yang J., Vais H., Gu W., Foskett J.K. (2016). Biphasic Regulation of Insp3 Receptor Gating by Dual Ca2+ Release Channel Bh3-Like Domains Mediates Bcl-Xl Control of Cell Viability. Proc. Natl. Acad. Sci. USA.

[90] Li C., Wang X., Vais H., Thompson C.B., Foskett J.K., White C. (2007). Apoptosis regulation by Bcl-xL modulation of mammalian inositol 1,4,5-trisphosphate receptor channel isoform gating. Proc. Natl. Acad. Sci. USA.

[91] Eckenrode E.F., Yang J., Velmurugan G.V., Foskett J.K., White C. (2010). Apoptosis Protection by Mcl-1 and Bcl-2 Modulation of Inositol 1,4,5-Trisphosphate Receptor-dependent Ca2+ Signaling. J. Biol. Chem..

[92] Minagawa N., Kruglov E.A., Dranoff J.A., Robert M.E., Gores G.J., Nathanson M.H. (2005). The Anti-apoptotic Protein Mcl-1 Inhibits Mitochondrial Ca2+ Signals. J. Biol. Chem..

[93] Puthalakath H., O’Reilly L.A., Gunn P., Lee L., Kelly P.N., Huntington N.D., Hughes P.D., Michalak E.M., McKimm-Breschkin J., Motoyama N. (2007). ER Stress Triggers Apoptosis by Activating BH3-Only Protein Bim. Cell.

[94] Pihán P., Carreras-Sureda A., Hetz C. (2017). BCL-2 family: Integrating stress responses at the ER to control cell demise. Cell Death Differ..

[95] Bonneau B., Ando H., Kawaai K., Hirose M., Takahashi-Iwanaga H., Mikoshiba K. (2016). IRBIT controls apoptosis by interacting with the Bcl-2 homolog, Bcl2l10, and by promoting ER-mitochondria contact. eLife.

[96] Vervliet T., Parys J.B., Bultynck G. (2016). Bcl-2 proteins and calcium signaling: Complexity beneath the surface. Oncogene.

[97] Vervliet T., Decrock E., Molgó J., Sorrentino V., Missiaen L., Leybaert L., De Smedt H., Kasri N.N., Parys J.B., Bultynck G. (2014). Bcl-2 binds to and inhibits ryanodine receptors. J. Cell Sci..

[98] Vervliet T., Lemmens I., Vandermarliere E., Decrock E., Ivanova H., Monaco G., Sorrentino V., Kasri N.N., Missiaen L., Martens L. (2015). Ryanodine receptors are targeted by anti-apoptotic Bcl-XL involving its BH4 domain and Lys87 from its BH3 domain. Sci. Rep..

[99] Zhivotovsky B., Orrenius S. (2011). Calcium and cell death mechanisms: A perspective from the cell death community. Cell Calcium.

[100] Danese A., Patergnani S., Bonora M., Wieckowski M.R., Previati M., Giorgi C., Pinton P. (2017). Calcium regulates cell death in cancer: Roles of the mitochondria and mitochondria-associated membranes (MAMs). Biochim. Biophys. Acta BBA Bioenerg..

[101] CsordásG G., Renken C., Várnai P., Walter L., Weaver D., Buttle K.F., Balla T., Mannella C.A., Hajnóczky G. (2006). Structural and functional features and significance of the physical linkage between ER and mitochondria. J. Cell Biol..

[102] Hwang M.-S., Schwall C.T., Pazarentzos E., Datler C., Alder N.N., Grimm S. (2014). Mitochondrial Ca2+ influx targets cardiolipin to disintegrate respiratory chain complex II for cell death induction. Cell Death Differ..

[103] Vervliet T., Clerix E., Seitaj B., Ivanova H., Monaco G., Bultynck G. (2017). Modulation of Ca2+ Signaling by Anti-apoptotic B-Cell Lymphoma 2 Proteins at the Endoplasmic Reticulum–Mitochondrial Interface. Front. Oncol..

[104] De Ridder I., Kerkhofs M., Veettil S.P., Dehaen W., Bultynck G. (2021). Cancer cell death strategies by targeting Bcl-2’s BH4 domain. Biochim. Biophys. Acta BBA Bioenerg..

[105] Zhong F., Harr M.W., Bultynck G., Monaco G., Parys J.B., De Smedt H., Rong Y.-P., Molitoris J.K., Lam M., Ryder C. (2011). Induction of Ca2+-driven apoptosis in chronic lymphocytic leukemia cells by peptide-mediated disruption of Bcl-2–IP3 receptor interaction. Blood.

[106] Akl H., Monaco G., La Rovere R., Welkenhuyzen K., Kiviluoto S., Vervliet T., Molgo J., Distelhorst C.W., Missiaen L., Mikoshiba K. (2013). IP3R2 levels dictate the apoptotic sensitivity of diffuse large B-cell lymphoma cells to an IP3R-derived peptide targeting the BH4 domain of Bcl-2. Cell Death Dis..

[107] Greenberg E.F., McColl K.S., Zhong F., Wildey G., Dowlati A., Distelhorst C.W. (2015). Synergistic killing of human small cell lung cancer cells by the Bcl-2-inositol 1,4,5-trisphosphate receptor disruptor BIRD-2 and the BH3-mimetic ABT-263. Cell Death Dis..

[108] Lavik A.R., Zhong F., Chang M.-J., Greenberg E., Choudhary Y., Smith M.R., McColl K.S., Pink J., Reu F.J., Matsuyama S. (2015). A synthetic peptide targeting the BH4 domain of Bcl-2 induces apoptosis in multiple myeloma and follicular lymphoma cells alone or in combination with agents targeting the BH3-binding pocket of Bcl-2. Oncotarget.

[109] Kerkhofs M., La Rovere R., Welkenhuysen K., Janssens A., Vandenberghe P., Madesh M., Parys J.B., Bultynck G. (2021). BIRD-2, a BH4-domain-targeting peptide of Bcl-2, provokes Bax/Bak-independent cell death in B-cell cancers through mitochondrial Ca2+-dependent mPTP opening. Cell Calcium.

[110] Schulman J.J., Wright F.A., Kaufmann T., Wojcikiewicz R.J.H. (2013). The Bcl-2 Protein Family Member Bok Binds to the Coupling Domain of Inositol 1,4,5-Trisphosphate Receptors and Protects Them from Proteolytic Cleavage. J. Biol. Chem..

[111] Schulman J.J., Wright F.A., Han X., Zluhan E.J., Szczesniak L.M., Wojcikiewicz R.J.H. (2016). The Stability and Expression Level of Bok Are Governed by Binding to Inositol 1,4,5-Trisphosphate Receptors. J. Biol. Chem..

[112] Schulman J.J., Szczesniak L.M., Bunker E.N., Nelson H.A., Roe M.W., Wagner L.E., Yule D.I., Wojcikiewicz R.J.H. (2019). Bok Regulates Mitochondrial Fusion and Morphology. Cell Death Differ..

[113] Nutt L.K., Pataer A., Pahler J., Fang B., Roth J., McConkey D.J., Swisher S.G. (2002). Bax and Bak Promote Apoptosis by Modulating Endoplasmic Reticular and Mitochondrial Ca2+ Stores. J. Biol. Chem..

[114] Scorrano L., Oakes S.A., Opferman J.T., Cheng E.H., Sorcinelli M.D., Pozzan T., Korsmeyer S.J. (2003). BAX and BAK Regulation of Endoplasmic Reticulum Ca2+: A Control Point for Apoptosis. Sci..

[115] Zong W.-X., Li C., Hatzivassiliou G., Lindsten T., Yu Q.-C., Yuan J., Thompson C.B. (2003). Bax and Bak can localize to the endoplasmic reticulum to initiate apoptosis. J. Cell Biol..

[116] Oakes S.A., Scorrano L., Opferman J.T., Bassik M.C., Nishino M., Pozzan T., Korsmeyer S.J. (2005). Proapoptotic BAX and BAK regulate the type 1 inositol trisphosphate receptor and calcium leak from the endoplasmic reticulum. Proc. Natl. Acad. Sci. USA.

[117] D’Orsi B., Kilbride S.M., Chen G., Alvarez S.P., Bonner H.P., Pfeiffer S., Plesnila N., Engel T., Henshall D.C., Düssmann H. (2015). Bax Regulates Neuronal Ca2+ Homeostasis. J. Neurosci..

[118] Wang X.E., Olberding K., White C., Li C. (2010). Bcl-2 proteins regulate ER membrane permeability to luminal proteins during ER stress-induced apoptosis. Cell Death Differ..

[119] Kanekura K., Ma X., Murphy J.T., Zhu L.J., Diwan A., Urano F. (2015). IRE1 prevents endoplasmic reticulum membrane permeabilization and cell death under pathological conditions. Sci. Signal..

[120] Salvador-Gallego R., Mund M., Cosentino K., Schneider J., Unsay J., Schraermeyer U., Engelhardt J., Ries J., García-Sáez A.J. (2016). Bax assembly into rings and arcs in apoptotic mitochondria is linked to membrane pores. EMBO J..

[121] Große L.A., Wurm C., Brüser C., Neumann D., Jans D.C., Jakobs S. (2016). Bax assembles into large ring-like structures remodeling the mitochondrial outer membrane in apoptosis. EMBO J..

[122] Hosoi K.-I., Miyata N., Mukai S., Furuki S., Okumoto K., Cheng E.H., Fujiki Y. (2017). The VDAC2–BAK axis regulates peroxisomal membrane permeability. J. Cell Biol..

[123] Csordás G., Madesh M., Antonsson B., Hajnóczky G. (2002). tcBid promotes Ca2+ signal propagation to the mitochondria: Control of Ca2+ permeation through the outer mitochondrial membrane. EMBO J..

[124] Klee M., Pallauf K., Alcalá S., Fleischer A., Pimentel-Muiños F.X. (2009). Mitochondrial apoptosis induced by BH3-only molecules in the exclusive presence of endoplasmic reticular Bak. EMBO J..

[125] Mathai J.P., Germain M., Shore G.C. (2005). BH3-only BIK Regulates BAX,BAK-dependent Release of Ca2+ from Endoplasmic Reticulum Stores and Mitochondrial Apoptosis during Stress-induced Cell Death. J. Biol. Chem..

[126] Mebratu Y.A., Leyva-Baca I., Wathelet M.G., Lacey N., Chand H.S., Choi A.M.K., Tesfaigzi Y. (2017). Bik reduces hyperplastic cells by increasing Bak and activating DAPk1 to juxtapose ER and mitochondria. Nat. Commun..

[127] Prevarskaya N., Skryma R., Shuba Y. (2011). Calcium in tumour metastasis: New roles for known actors. Nat. Rev. Cancer.

[128] Clapham D.E. (2007). Calcium Signaling. Cell.

[129] Prudent J., Popgeorgiev N., Gadet R., Deygas M., Rimokh R., Gillet G. (2016). Mitochondrial Ca2+ uptake controls actin cytoskeleton dynamics during cell migration. Sci. Rep..

[130] Tosatto A., Sommaggio R., Kummerow C., Bentham R.B., Blacker T.S., Berecz T., Duchen M.R., Rosato A., Bogeski I., Szabadkai G. (2016). The mitochondrial calcium uniporter regulates breast cancer progression via HIF -1α. EMBO Mol. Med..

[131] Ishikawa K., Koshikawa N., Takenaga K., Nakada K., Hayashi J.-I. (2008). Reversible regulation of metastasis by ROS-generating mtDNA mutations. Mitochondrion.

[132] Ishikawa K., Takenaga K., Akimoto M., Koshikawa N., Yamaguchi A., Imanishi H., Nakada K., Honma Y., Hayashi J.-I. (2008). ROS-Generating Mitochondrial DNA Mutations Can Regulate Tumor Cell Metastasis. Science.

[133] Bessou M.J., Lopez R., Gadet M., Deygas N., Popgeorgiev D., Poncet A., Nougarede P., Billard I., Mikaelian P., Gonzalo R. (2020). The Apoptosis Inhibitor Bcl-Xl Controls Breast Cancer Cell Migration through Mitochondria-Dependent Reactive Oxygen Species Production. Oncogene.

[134] Fouqué A., Lepvrier E., Debure L., Gouriou Y., Malleter M., Delcroix V., Ovize M., Ducret T., Li C., Hammadi M. (2016). The apoptotic members CD95, BclxL, and Bcl-2 cooperate to promote cell migration by inducing Ca(2+) flux from the endoplasmic reticulum to mitochondria. Cell Death Differ..

